# Low Host Specialization in the Cuckoo Wasp, *Parnopes grandior*, Weakens Chemical Mimicry but Does Not Lead to Local Adaption

**DOI:** 10.3390/insects11020136

**Published:** 2020-02-20

**Authors:** Carlo Polidori, Yolanda Ballesteros, Mareike Wurdack, Josep Daniel Asís, José Tormos, Laura Baños-Picón, Thomas Schmitt

**Affiliations:** 1 Institute of Environmental Sciences (ICAM), University of Castilla La Mancha. Avda, Carlos III s/n, 45071 Toledo, Spain; 2 Zoology Unit, Faculty of Biology, University of Salamanca, 37071 Salamanca, Spain; sandwasp@outlook.com (Y.B.); asis@usal.es (J.D.A.); tormos@usal.es (J.T.); lbanos@usal.es (L.B.-P.); 3 Department of Animal Ecology and Tropical Biology, Biocenter, University of Würzburg, Am Hubland, 97074 Würzburg, Germany; mareike.wurdack@gmail.com (M.W.); thomas.schmitt@uni-wuerzburg.de (T.S.)

**Keywords:** Chrysididae, *Bembix*, chemical mimicry, cuticular hydrocarbons

## Abstract

Insect brood parasites have evolved a variety of strategies to avoid being detected by their hosts. Few previous studies on cuckoo wasps (Hymenoptera: Chrysididae), which are natural enemies of solitary wasps and bees, have shown that chemical mimicry, i.e., the biosynthesis of cuticular hydrocarbons (CHC) that match the host profile, evolved in several species. However, mimicry was not detected in all investigated host-parasite pairs. The effect of host range as a second factor that may play a role in evolution of mimicry has been neglected, since all previous studies were carried out on host specialists and at nesting sites where only one host species occurred. Here we studied the cuckoo wasp *Parnopes grandior*, which attacks many digger wasp species of the genus *Bembix* (Hymenoptera: Crabronidae). Given its weak host specialization, *P. grandior* may either locally adapt by increasing mimicry precision to only one of the sympatric hosts or it may evolve chemical insignificance by reducing the CHC profile complexity and/or CHCs amounts. At a study site harbouring three host species, we found evidence for a weak but appreciable chemical deception strategy in *P. grandior*. Indeed, the CHC profile of *P. grandior* was more similar to all sympatric *Bembix* species than to a non-host wasp species belonging to the same tribe as *Bembix*. Furthermore, *P. grandior* CHC profile was equally distant to all the hosts’ CHC profiles, thus not pointing towards local adaptation of the CHC profile to one of the hosts’ profile. We conducted behavioural assays suggesting that such weak mimicry is sufficient to reduce host aggression, even in absence of an insignificance strategy, which was not detected. Hence, we finally concluded that host range may indeed play a role in shaping the level of chemical mimicry in cuckoo wasps.

## 1. Introduction

Females of many insect brood parasites sneak into the host nests in order to deposit their eggs or larvae, a behaviour allowing parasites to easily leave chemical traces in the nests. These cues may provoke a defensive response by the hosts, i.e., the abandonment of the nest, the destruction of the parasites’ eggs or direct attack to the adult parasite [1,2,3]. Thus, to reduce the probability of being detected by their hosts during nest invasion, insect brood parasites have evolved different strategies that often prevent recognition via chemical cues [4,5,6,7]. These strategies were particularly studied in aculeate Hymenoptera (bees, wasps and ants), a diverse insect group in which parasitism evolved independently many times [8,9,10]. Within the Aculeata, brood parasitoids (females laying eggs on or into the host immatures), kleptoparasites (females laying eggs on the host food resources) and social parasites (reproductive females invading a social host nest and exploiting the worker force of the host colony for breeding) occur [6,8,9,10].

Chemical strategies to successfully avoid host aggression during nest invasion are common in parasitic aculeate Hymenoptera. They can be grouped into three main categories: (a) chemical mimicry, which occurs when a parasite synthetises *de novo* an odour bouquet matching that of the host’s bouquet [11], (b) chemical camouflage, which occurs when the host odour bouquet is acquired from the host [9] and (c) chemical insignificance, which occurs when the brood parasites have reduced recognition cues, which limit their chance of being perceived by the hosts [12]. All these strategies might have an impact on the evolution of cuticular hydrocarbon (CHC) profiles, i.e., the thin layers of a complex mixture of non-polar substances that covers the whole external surface of insects. Indeed, while the primary function of CHCs is to reduce desiccation, abrasion or infection, they also commonly act as semiochemicals in different contexts of communication, including intra-specific and inter-specific recognition [7,13].

Although these strategies were detected in parasitic aculeate Hymenoptera, it is still unclear which factors may promote such alternative adaptations in an evolutionary context. For example, chemical camouflage was frequently found as a strategy in socially parasitic wasps and ants and to a limited extent in socially parasitic bees [6,9,14,15,16]. Insignificance was observed in many social parasites, too, as parasites tend to show a reduced number of cues at the nest invasion stage, prior to colony integration [6,9], as well as in few solitary brood parasites [12]. On the other hand, mimicry was observed only in few species within the family Chrysididae (cuckoo wasps) [11,17], which include parasitoids or kleptoparasites of wasps and bees [18]. In particular, precise chemical mimicry was recognised in *Hedychrum rutilans* Dahlbom, 1854, a natural enemy of the wasp *Philanthus triangulum* (Fabricius, 1775) and to a lesser extent of *Philanthus coronatus* (Thunberg, 1784), and in *Chrysis mediata* Linsenmaier, 1951 and *Pseudospinolia neglecta* (Shuckard, 1837), each attacking only one out of two different chemotypes of the eumenine wasp *Odynerus spinipes* (Linnaeus,1758) [11,17]. All these cuckoo wasp species enter active, open host nests, making chemical mimicry essential to avoid being detected. However, chemical mimicry was not found in *Chrysis viridula* Linnaeus, 1761. Females of this species re-open already sealed nests of *O*. *spinipes* to oviposit [17]. Additionally, evidence for a less precise mimicry was found in *Hedychrum nobile* (Scopoli, 1763), which attacks several species of the wasp genus *Cerceris* [11]. Interestingly, the few cuckoo wasp species for which a strong chemical mimicry was detected are all highly specialised in the host choice, suggesting that host range may play a role in shaping chemical strategies. However, strategies that generalist cuckoo wasps may adopt in the presence of several sympatric hosts have not been investigated yet.

Here, we studied the cuckoo wasp, *Parnopes grandior* (Pallas, 1771). At least 10 *Bembix* (Hymenoptera: Crabronidae) species are recorded as hosts of this cuckoo wasp [2,19,20,21,22,23,24,25], suggesting that it is a brood parasitoid specialist at the genus level. *Bembix* wasps are Diptera-hunting predators that nest in aggregations in sandy soil, where *P. grandior* often co-occurs [26]. We analysed host-parasite relationships at a Spanish site where three *Bembix* species (*Bembix merceti* Parker 1904, *Bembix sinuata* Panzer 1804 and *Bembix zonata* Klug 1835) occur. Host relationships between *P. grandior* and all of these species except from *B. merceti* were confirmed by observing cuckoo wasps emerging from the wasp brood cells [21,24]. Furthermore, *P. grandior* also visits *B. merceti* nests (Polidori et al. unpublished data), even sometimes fighting outside nests (J. T, pers. observation). Thus, we consider this species as a potential host. *Bembix* females frequently bring prey items to the nest during a large part of brood development and check larval growth until it is fully provisioned. *Parnopes grandior* females oviposit in the host nests during these provisioning flights of host females [24,25]. While host females are able to detect eggs of *P. grandior*, and, in consequence, abandon their nests [2], cuckoo wasps would be favoured to chemically mimicking their hosts, preventing them from leaving chemical cues.

Because of its low host specialization, however, *P. grandior* would not be able to chemically match all available hosts at a given location, given that CHC profiles tend to be species-specific in insects [7,13]. In particular, we might document local adaptation to one of the host species as shown in other insect brood parasites, including socially parasitic ants, bee-parasitic beetles and ant-parasitic Lepidoptera [27,28,29]. Alternatively, *P. grandior* may maintain a weak mimicry to all present hosts and/or reduce the CHC profile complexity and/or CHC amounts (insignificance strategy). The latter strategy was also previously observed in parasitic Hymenoptera [30].

We thus tested two alternative hypotheses. The first hypothesis is that the CHC profile of *P. grandior* weakly overlaps with the CHC profile of any host species (low level of chemical mimicry) and/or presents a less complex profile and/or lower amounts of CHCs (chemical insignificance). The second hypothesis is that the *P. grandior* population is chemically more similar to just one of the three *Bembix* species, a strategy that could be explained by local adaptation. To test these hypotheses, we compared the CHCs profile of *Bembix* spp., *P. grandior* and a non-host “outgroup” species (*Stizus continuus* (Klug, 1835) belonging to the same tribe as *Bembix* (Bembicini) [31]. Furthermore, we performed behavioural assays to investigate whether the hosts can recognise the CHC profile of *P. grandior*. Finally, in host-parasite arms races, higher parasite prevalence and fitness costs to the hosts may increase recognition ability of the hosts, in turn leading to a higher precision of chemical mimicry in the parasite as a counter adaptation [32,33]. Therefore, we collected data on parasitism rates to evaluate its significance for chemical and behavioural differences.

## 2. Materials and Methods

### 2.1. Study Area and Sample Collection

Field work was carried out at a nesting area of *Bembix* species nearby the small town of Almarail (province of Soria, Spain) (41°34′50″ N 2°22′52″ W, 978 m of altitude) in July 2011–2013. Females of the non-host species *S. continuus* were collected at a saline-sandy soil nesting site nearby the small town El Saler (Province of Valencia, Spain) (39°22′57″ N 0°19′57″ W, 3 m of altitude) in June–August 2010 [34]. *Bembix merceti*, *B. sinuata* and *B. zonata* nested in an area of about 4 km^2^ with overlapping nesting sites [35]. At this location cereal crops with small patches of woodland dominate the landscape. *Bembix* species nest in old fallow plots [35]. Individuals of *Bembix* spp. and *P. grandior* were weighted in the field upon collection with an Ohaus Scout Pro balance to the nearest 0.002 g. Five females *per* species were collected and analysed. Upon collection, these 25 individuals were frozen and stored at −20 °C for the subsequent chemical analyses. A total of 22, 20 and 30 brood cells of, respectively, *B. merceti*, *B. zonata* and *B. sinuata* were excavated at the end of summer 2011 and were kept individually in boxes at 6 °C until next spring. Once extracted from the fridge, individuals were allowed to eclose. Rate of parasitism *per* host species was calculated as number of parasitised cells divided by the number of excavated cells.

### 2.2. Behavioural Experiments

We carried out behavioural assays to test if *Bembix* females are able to recognise *P. grandior* as a foe and behaves accordingly, i.e., being aggressive. Circle tube experiments were carried out at the hosts’ nesting site, placing a parasite and a host (one female of either *B. merceti*, *B. sinuata* or *B. zonata* and one female of *P. grandior*) in a 45 cm-long, 1 cm-wide transparent silicon tube. [36,37,38]. Host wasps were always allowed to enter first in order to mimic the situation of a host wasp staying in its nest while a cuckoo wasp is entering it. We used each tube only once to avoid odour contamination by previous occupants [39].

Behavioural tests were performed between 900 h and 1500 h during the foraging period of hosts and cuckoo wasps. Host females were collected while exiting from or returning to their nests. Cuckoo wasp females were collected while patrolling the nest aggregations or while trying to enter a nest. Individuals were colour marked on the thorax and released after the experiments, so that no individuals were used in more than one trial. Eight to 12 trials were performed per each of the three species pairs (for a total of 64 tested individuals). While we kept the individuals in the tubes for 15 min, which is a standard duration for circle-tube experiments [36,37,38], we noted that the activity decreased or even stopped after 5 to 7 min. Thus, we assessed the first five interactions and their rate (number/time). Aggressive, tolerant and avoidance behaviours were considered. Aggressive interactions occurred when a female curls her abdomen under the thorax with the intention to sting the other female, or when a female clamps the mandibles around the neck, limbs or antenna of the other female. The tolerant interactions include the close-up, non-aggressive contacts, such as accommodating their bodies and passing in opposite directions or stopping at a short distance frontally and gently antennating each other. Finally, the avoidance interactions typically include turn-around movements or backing movements without reverse after a frontal encounter, while increasing distances. All these behaviours and their categorization were defined according with previous studies in Apoidea [36,37,38]. Behavioural data are available in the Supplementary Table S1.

### 2.3. Characterization of the Cuticular Hydrocarbon Profile (CHC)

To extract cuticular hydrocarbons individuals were allowed to thaw for two minutes. The specimens were immersed in a sufficient amount of *n*-hexane to cover the entire body for 10 min. 1000 ng of C18 as an internal standard was added prior to the extraction process. Extracts were concentrated with a gentle stream of N_2_ to approximately 80–100 µL remained and stored at −20 °C. The specimens were stored in 95% ethanol and their species identification confirmed. We processed the extracts with a HP 6890 gas chromatograph (GC) coupled to a HP 5973 Mass Selective Detector (MS) (Hewlett Packard, Waldbronn, Germany) or an Agilent 7890/5975 GCMS System. The GC (split/splitless injector in splitless mode for 1 min, injected volume: 1 µl at 300 °C injector temperature) was equipped with a DB-5 Fused Silica capillary column (30 m x 0.25 mm ID, df = 0.25 µm, J&W Scientific, Folsom, USA). Helium was used as carrier gas with a constant flow of 1 mL/min. For both GC/MS, the temperature program starts at 60 °C with a subsequent increase of 5 °C/min until 300 °C and kept isotherm at 300 °C for 10 min. An ionization voltage of 70 eV (source temperature: 230 °C) was set for the acquisition of the mass spectra by electron ionization (EI-MS).

The software MSD ChemStation G1701EA E.02.02.1431 was used to record and analyse the chromatograms and mass spectra. The MS data base Wiley275 (John Wiley & Sons, New York, USA), the compound-specific retention time, Kovats indices, and the detected diagnostic ions [40] were used to chemically identify CHC compounds. For few substances eluting at similar retention times, we combined these compounds and classified them as blends.

Once all peaks were quantified, we omitted all compounds that added less than 0.01% to the overall relative amount within each species. However, if a compound contributed more than 0.01% (average across individuals) in a single species, we kept it in all investigated species for the comparative analysis. In a second step, we eliminated all compounds that did not occur at least in 50% of all individuals within a species. Yet again, if a compound occurs in more than half of the individuals of a single species, we kept it in all species. Chemical data are available in the Supplementary Table S2.

### 2.4. Statistical Analysis

To analyse the behavioural data, we first calculated the median number of interactions *per* minute across trials, for each of the three behavioural responses (aggressive, tolerant and avoidance), occurring in each species pair. Then, we tested for differences among behavioural responses with the nonparametric Kruskal-Wallis test, followed by post-hoc Dunn’s test for pairwise comparisons [41]. The *p*-values obtained from Dunn’s procedure were the Bonferroni corrected values. The same test was used to verify if the proportion of the different classes of hydrocarbons (linear alkanes, alkenes, mono- and dimethyl-branched alkanes and alkadienes) differed among species. Differences between the rates of parasitism of *Bembix* spp. were tested with the Z-test.

The final matrix of the chemical data included 106 peaks (Appendix A). Prior to the statistical analysis of the chemical data, we transformed all the peak values, to avoid undefined values for peaks with an area of zero, as log_10_((relative peak area/geometric mean of relative peak area)+1) [11].

To test for chemical mimicry in *P. grandior*, we performed a series of multivariate analyses, all based on a Bray–Curtis dissimilarity matrix, which is suitable for zero-inflated datasets [42]. All these analyses do not require *a priori* grouping of species, meaning that these methods allow pattern formation that are exclusively based on CHC similarities.

First, we performed an agglomerative cluster analysis based on the unweighted pair group method using arithmetic means of Bray–Curtis dissimilarities [42]. Second, we created a network plot, in which nodes (individuals) are connected by edges, with the diameter of nodes proportional to the number of edges connected to it, and the thickness of edges proportional to the CHC profile similarity. In this representation of similarities among individuals of all species, we chose a 50% similarity cut-off to control the number of edges and increase clarity (i.e., only edges between nodes with more than 50% similarity are shown) [43]. Third, Bray-Curtis dissimilarities were used for ordinations using non-metric multidimensional scaling analysis (NMDS), which is a non-parametric method that avoids assuming linearity among variables [44] and whose resulting plot shows the spatial distances between individuals (i.e., their chemical distances). In the NMDS, deviations are expressed in terms of “stress”, for which values ≤ 0.15 indicate a good fit of ordination [45]. PERMANOVA (Non-Parametric MANOVA (Multivariate Analysis of Variance) was employed to test for differences among the studied species [46]. The significance is computed by permutation of group membership (9999 replicates). Pairwise PERMANOVA between all pairs of groups was also computed as a post-hoc test. Similarity percentages (SIMPER) were calculated to identify the compounds that predominantly contributed to the Bray-Curtis dissimilarities among all species [47]. SIMPER also provides the dissimilarity values between all pairs of species. We used it, together with the pairwise PERMANOVA, to evaluate how similar the CHC profiles of the cuckoo wasps are in comparison to their hosts.

To verify if cuckoo wasps are chemically insignificant to their hosts, we compared two chemical traits among species. First, we compared the total number of peaks among species as an indication of CHC profile complexity [29]. Second, we compared the overall amount of all CHCs as the sum of all peak areas relative to the area of the linear alkane C18, by correcting for insect body weight (ng of hydrocarbons/mg of insect body weight). These comparisons were tested with the Kruskal-Wallis test, followed by post-hoc Dunn’s test for pairwise comparisons (Bonferroni corrected *p*-values).

The statistical analyses were performed in XLSTAT 2008 and in PAST 3.04 (Paleontological Statistics Software Package) [48].

## 3. Results

### 3.1. Behavioural Interactions

**Figure 1:**
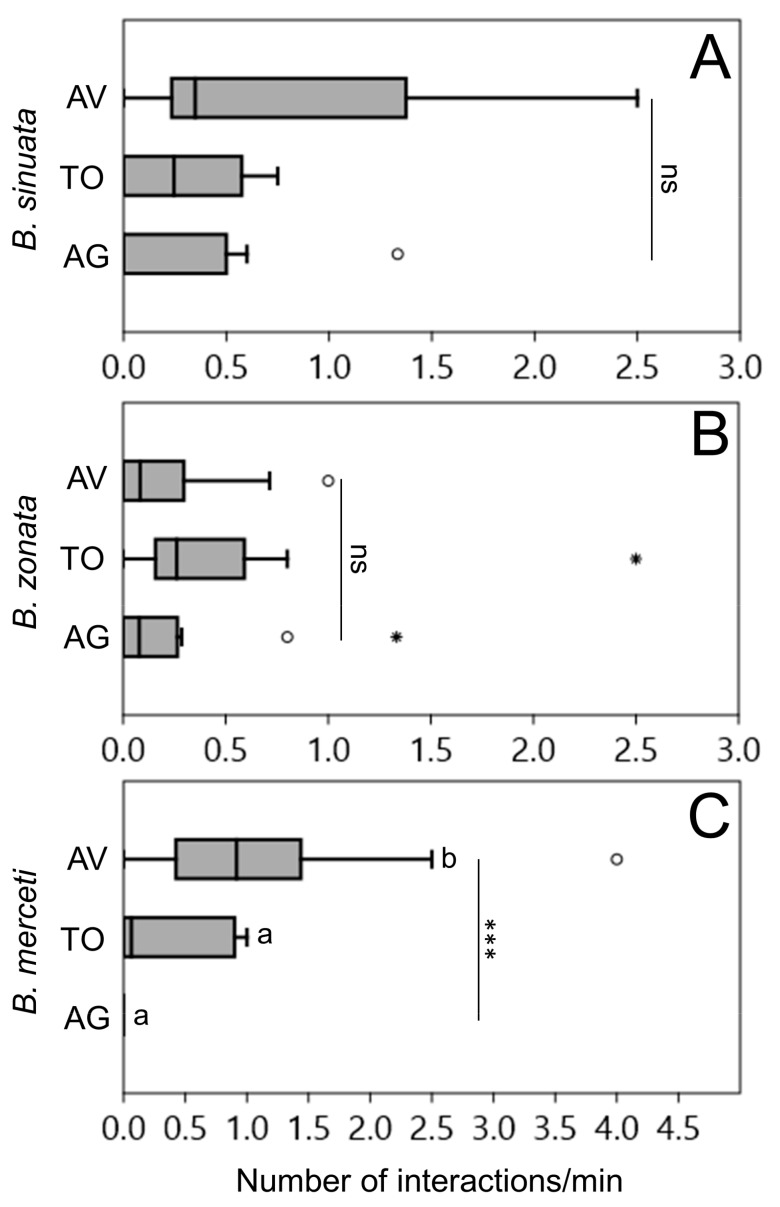
Box-and-whisker plots showing medians (horizontal lines within boxes), 1° and 3° quartile (horizontal lines closing the boxes), and maximum and minimum values (ends of the whiskers) for the number of behavioral interactions/minute recorded in circle-tube experiments between the cuckoo wasp *P. grandior* and its three hosts *B. sinuata* (**A**), *B. zonata* (**B**) and *B. merceti* (**C**). Outliers with a value more than 1.5 times the interquartile range are shown as circles, values with more than three times the interquartile range are shown as stars. *** means that differences among types of interactions are significant at *p* < 0.001; letters identify pairwise differences (Dunn’s procedure). AV = avoidance interactions, TO = tolerant interactions, AGG = aggressive interactions.

The three tested *Bembix* species showed differences in their responses to the presence of *P. grandior* in the circle tubes. *B. zonata* was the species that performed the lowest number of interactions per minute (0.7), while the other two species showed similar numbers of interactions with the cuckoo wasp per minute (1–1.1). Most of the recorded interactions involved avoidance behaviour in *B. sinuata* (median per minute across trials: 0.35) (Figure 1A) and *B. merceti* (0.92) (Figure 1C), while less avoidance behaviour was observed in *B. zonata* (0.08) (Figure 1B). In the latter species, the highest frequency of tolerant behaviour has been recorded (median per minute across trials: 0.26) (Figure 1B), followed by *B. sinuata* (0.24) (Figure 1A) and by *B. merceti* (Figure 1C), in which tolerant behaviour was very rare (0.6). All three species of *Bembix* showed zero (*B. merceti*) or low levels of aggression towards the cuckoo wasp (medians per minute across trials: 0.00–0.03) (Figure 1).

Kruskal-Wallis tests showed no significant differences among median values of aggressive, tolerant and avoidance interactions in *B. sinuata* (χ^2^ = 2.53, n = 16, *p* = 0.26) and *B. zonata* (χ^2^ = 2.98, n = 24, *p* = 0.20), while a difference exists in *B. merceti* (χ^2^ = 16.68, n = 24, *p* < 0.0001), where avoidance interactions were significantly more frequent (Dunn’s test with Bonferroni corrected *p*-values: avoidance vs. tolerant: *p* = 0.04, avoidance vs. aggressive: *p* < 0.0001, aggressive vs. tolerant: *p* = 0.13). The three host species did not differ in their aggression levels (χ^2^ = 2.97, n = 16, *p* = 0.10) (Figure 1).

Rate of parasitism was 0% in *B. merceti* (n = 22), 15% in *B. zonata* (n = 20) and 23.3% in *B. sinuata* (n = 30). These values were statistically similar (Z < −1.8, *p* > 0.06), except for *B. merceti*, which had a lower parasitism rate than *B. sinuata* (Z = −2.4, *p* = 0.013).

### 3.2. Characterization of CHC Profiles

**Figure 2:**
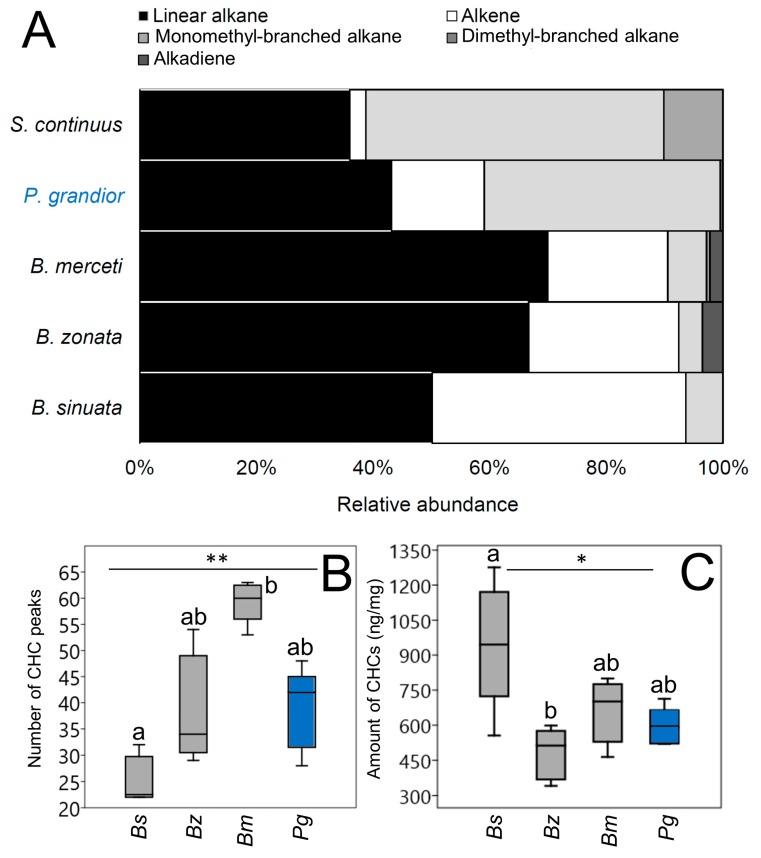
(**A**) Relative amount (in %) of the hydrocarbons substance classes in the cuticular hydrocarbon (CHC) profiles of *P. grandior* (in blue), its *Bembix* hosts and the non-host *S. continuus* (all in black); (**B**) Box-and-whisker plots showing medians (horizontal lines within boxes), 1° and 3° quartile (horizontal lines closing the boxes) and maximum and minimum values (ends of the whiskers) for the number of CHC peaks occurring in *P. grandior* and its *Bembix* hosts; (**C**) Box-and-whisker plots showing medians (horizontal lines within boxes), 1° and 3° quartile (horizontal lines closing the boxes) and maximum and minimum values (ends of the whiskers) for the amount of CHCs (nm/mg) on the cuticle of *P. grandior* and its *Bembix* hosts. * and ** mean that differences among groups are significant at *p* < 0.05 and *p* < 0.01, respectively; letters identify pairwise differences (Dunn’s procedure). Bs = *B. sinuata*, Bz = *B. zonata*, Bm = *B. merceti*, Pg = *P. grandior*.

Linear alkanes, monomethyl-branched alkanes, dimethyl-branched alkanes, alkenes and alkadienes with chain lengths ranging from 20 to 35 carbon atoms occurred as main components on the cuticle of all studied species (Appendix A, Figure 2A). Overall, linear alkanes dominated the CHC profiles of all studied species (43–70% of relative amount), though with differences (Kruskal-Wallis test, χ^2^ = 20.31, n = 25, *p* = 0.0004). In particular, in paired comparisons, *Bembix* spp. did not differ in their relative amount of linear alkanes, while *B. zonata* and *B. merceti* had higher proportions of linear alkanes than *S. continuus*, and *B. merceti* had higher proportions of linear alkanes than *P. grandior* (Dunn’s test with Bonferroni corrected *p*-values: *p* = 0.002-0.03) (Appendix A, Figure 2A). Dimethyl-branched alkanes were generally rare, occurring in lower proportions (0.60%) in *B. merceti* and in a higher proportion (10.1%) in *S. continuus* (Kruskal-Wallis test, χ^2^ = 18.46, n = 25, *p* < 0.0001). *S. continuus* had a significantly higher relative amount of dimethyl-branched alkanes than the other species (Dunn’s test with Bonferroni corrected *p* -values: *p* = 0.002) (Appendix A, Figure 2A) (Appendix A, Figure 2A). Alkadienes were also rare, not representing more than 3.6% of the CHC composition, and their proportions differed among species (Kruskal-Wallis test, χ^2^ = 13.39, n = 25, *p* = 0.003); *B. merceti* had in particular a higher relative amount of alkadienes than *B. sinuata* (Dunn’s test with Bonferroni corrected *p* -value: *p* = 0.013). The relative amount of alkenes showed a large variation among species (Kruskal-Wallis test, χ^2^ = 21.61, n = 25, *p* = 0.0002). Notably, all *Bembix* and *P. grandior* have > 15% of alkenes, while *S. continuus* has only 2.8%. However, the non-host species has significantly lower proportion of alkenes only compared with *B. sinuata* and *B. zonata* (Dunn’s test with Bonferroni corrected *p* -values: *p* = 0.0001–0.017). Additionally, *P. grandior* has a lower relative amount of alkenes than *B. sinuata* (Dunn’s test with Bonferroni corrected *p*-value: *p* = 0.04) (Appendix A, Figure 2A). Monomethyl-branched alkanes were less abundant (< 7%) in the CHC profile of *Bembix*, while they exhibit high proportions in *P. grandior* and in *S. continuus* (> 40%) (Kruskal-Wallis test, χ^2^ = 19.72, n = 25, *p* =0.0005; Dunn’s test with Bonferroni corrected *p*-values: *p* = 0.001-0.015) (Appendix A, Figure 2A).

The total number of CHC peaks per species, which overall ranged from 32 (*B. sinuata*) to 67 (*B. merceti*), differed significantly among species (Kruskal-Wallis test, χ^2^ = 13.41, n = 25, *p* = 0.003), with *B. merceti* having the highest number of peaks (Dunn’s test with Bonferroni corrected *p* -values: *p* = 0.002). All other pairwise comparisons, including those between *P. grandior* (50 peaks) and its hosts (Dunn’s test with Bonferroni corrected *p* -values: *p* = 0.15–1), were not significant (Figure 2B). The total amount of CHCs relative to body mass (ng/mg) also differed significantly among species (Kruskal-Wallis test, χ^2^ = 9.65, n = 20, *p* = 0.02), with *B. sinuata* having the highest amount (median = 945 ng/mg) (medians = 512–701 ng/mg) (Dunn’s test with Bonferroni corrected *p*-values: *p* = 0.013). All the other species presented similar amounts (Dunn’s test with Bonferroni corrected *p*-values: *p* = 0.32–1) (Figure 2C). Out of the 50 peaks occurring on *P. grandior*, 22 (mostly linear alkanes) also occurred in all the host species. However, only one of the peaks occurring in *P. grandior*, a rare monomethyl-branched alkane accounting only for the 0.03% of the CHC profile, was absent from all *Bembix* spp., i.e., *P. grandior* does not possess high amounts of exclusive compounds. On the contrary, *P. grandior* possesses many substances (21) that lacked in the non-host species, *S. continuus*.

**Figure 3:**
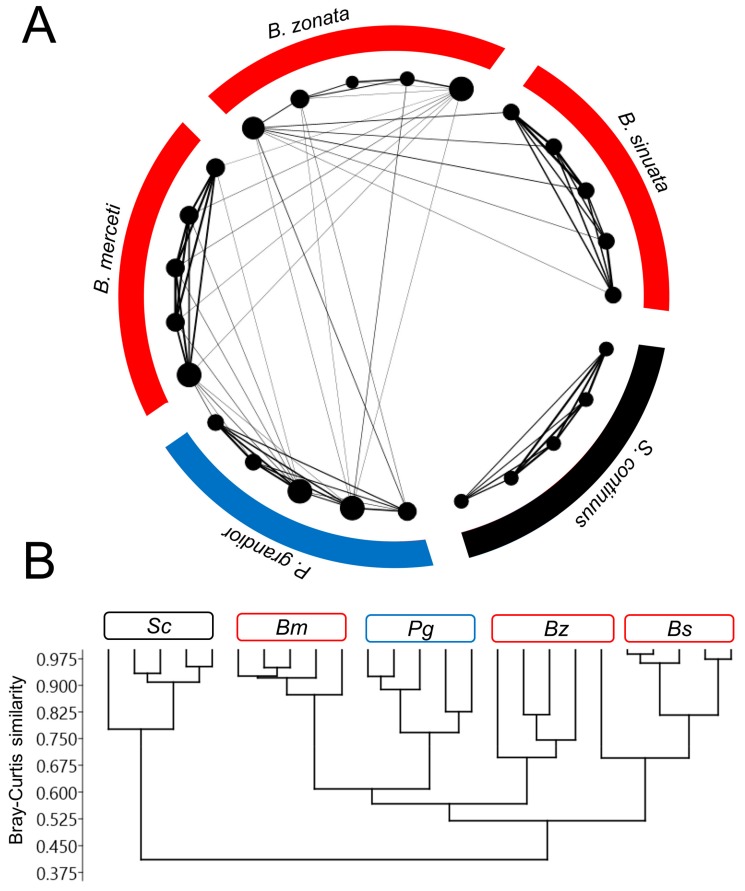
(**A**) Network plot based on Bray-Curtis distances showing the similarities among all individual CHC profiles of *P. grandior*, its *Bembix* hosts, and the non-host *S. continuus*. Only edges connecting individuals (i.e., nodes) with > 50% similarity in their CHC profiles are shown. The diameter of nodes is proportional to the number of edges connected to it, and the thickness of edges is proportional to the similarity. (**B**) Dendrogram based on the agglomerative cluster analysis (Bray-Curtis distances) of all individual CHC profiles of *P. grandior*, its *Bembix* hosts, and the non-host *S. continuus*. Bs = *B. sinuata*, Bz = *B. zonata*, Bm = *B. merceti*, Pg = *P. grandior*, Sc_S = *S. continuus*.

A network plot (Figure 3A) and a cluster analysis (Figure 3B) showed that the *Bembix* species have more similar CHC profiles (in terms of Bray-Curtis distances) among them and with *P. grandior*, compared with the much weaker connections (i.e., higher dissimilarities) with *S. continuus*. In the network plot, the 50% similarity cut-off even isolated *S. continuus* from all the other species (no connections shown) (Figure 3A), while in the cluster analysis *S. continuus* constitutes the group with the most distant CHC profile (first bifurcation) (Figure 3B). In the cluster analysis, a second bifurcation separated *B. merceti*, *B. zonata* (all individuals except one) and *P. grandior* from *B. sinuata* (and the remaining individual of *B. zonata*), while the third bifurcation separated *B. zonata* from *P. grandior* + *B. merceti* (Figure 3B). Thus, *Bembix* spp. were overall chemically more similar to *P. grandior* than to the phylogenetically closer digger wasp species *S. continuus*.

**Figure 4:**
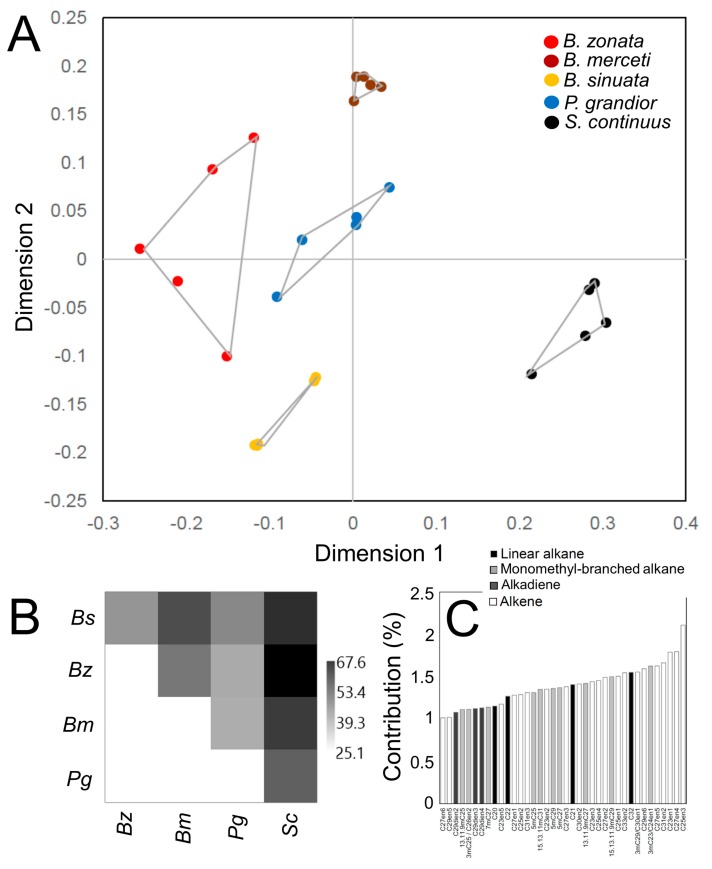
(**A**) Non-metric multidimensional scaling (NMDS) based on Bray-Curtis distances of all individual CHC profiles of *P. grandior*, its *Bembix* hosts, and the non-host *S. continuus*. (**B**) Matrix plot showing SIMPER (similarity percentages) dissimilarities among all the studies species. (**C**) Histogram showing all substances with > 1% contribution to the SIMPER dissimilarity among the studied species and their relative amount contributing to CHC profiles; white, light grey, dark grey and black bars correspond to substances of different groups (alkenes, mono- and dimethyl-branched alkanes, alkadienes and linear alkanes). Bs = *B. sinuata*, Bz = *B. zonata*, Bm = *B. merceti*, Pg = *P. grandior*, Sc = *S. continuus*.

The NMDS revealed species-specific CHC profiles (stress = 0.15) (PERMANOVA: F = 27.82, total sum of squares = 2.74, within-group sum of squares = 0.41, *p* < 0.0001) (Figure 4A). Indeed, all pairwise PERMANOVA tests were significant (F = 10.12–94.15, *p* < 0.01). The SIMPER analysis showed that pairwise distances were highest between *S. continuus* and all the other species (57.3–67.6), while lower distances were found among *Bembix* species (43.0–54.5) and among *P. grandior* and its hosts (39.1–45.1) (Figure 4B). The SIMPER analysis revealed that the substances contributing to more than 1% of CHCs dissimilarities among species (38 substances for a total of 52.7% of contribution) were alkenes (20 substances for a total of 29.2% of contribution), followed by monomethyl-branched alkanes (11 substances for a total of 14.8% of contribution) (Figure 4C). Alkadienes and linear alkanes were less important (3–4 substances and 3.3–5.3% of contribution, respectively) (Figure 4C). Dimethyl-branched alkanes were not important (<1% contribution).

## 4. Discussion

It is likely that the dependence of insect brood parasites on their hosts imposes selection on the latter to avoid being recognised by the hosts, and such selection often involves the modification of host recognition cues, such as CHCs [6,11,49,50]. Such modification may involve chemical mimicry, chemical insignificance or chemical camouflage. Our study aimed to investigate which chemical strategy may be adopted by *P. grandior*, a cuckoo wasp species that is known to attack several host species even at a single nesting site [34], and, hence, may be limited in precisely matching its CHC profile with those of the hosts. In fact, CHC profiles are often species-specific in insects [13,47]. Indeed, while many parasites specialise in a single or very few host species and consequently closely adapt their behaviour, morphology and chemistry to them [51,52,53], more generalist parasites face a trade-off. For example, the adaptation to one of the available hosts (i.e., local adaptation by CHC matching with one host) may entail costs for the interactions with the other hosts [54]. On the other hand, using different hosts may entail costs related with a weaker deception strategy, i.e., CHC matching may be weak to all available hosts, and consequently, parasites may be recognised and counter-attacked by their hosts. The latter strategy may, however, be facilitated by reducing in parallel the CHC profile complexity and/or amount (insignificance) [30]. Here, we provide evidence that *P. grandior* evolved a weak but appreciable chemical mimicry strategy, albeit not in conjunction with an insignificant strategy, and that local adaptation seems unlikely.

While both quantitative and qualitative differences were found among the studied species, our data revealed that the CHC profile of *P. grandior* has a shorter chemical distance to all of its *Bembix* hosts studied compared with the larger chemical distance observed to a non-host species, *S. continuus*, which is also from the tribe Bembicini. This strongly suggests that the cuckoo wasp chemically matches all the host species to a certain extent. In particular, *P. grandior* overall presents a CHC profile that almost does not include exclusive compounds. While cuckoo wasps lacked many compounds found in the hosts, they do not possess compounds lacking in all hosts, except one rare monomethyl-branched alkane. On the other hand, many compounds found in the cuckoo wasps are not present in *S. continuus*. The main differences in the CHC profile among the studied species largely rely on alkenes, a class of hydrocarbons known to be important in communication contexts, including intra- and interspecific recognition [55,56]. The inclusion of *S. continuus* in our analysis as “outgroup” species was thus important to reveal patterns of mimicry. Similar results were reported by a study on the chemical strategy of another cuckoo wasp and its host. While studying chemical mimicry of *H. rutilans* towards its host *P. triangulum*, the inclusion of the non-host but closely related species *Cerceris arenaria* (Linnaeus,1758) helped to reject the hypothesis that closer phylogenetic relationships drive closer chemical resemblances between species [11]. We could not include a second species of Chrysididae as outgroup to test the hypothesis that, in case of mimicry, *P. grandior* would show a larger chemical distance to a closely related cuckoo wasp than to its hosts, as we had no access to other species within the tribe Parnopini. However, by inspecting the CHC profiles of the cuckoo wasp species studied thus far [11,17], large differences appear compared to *P. grandior*. In particular, all the other cuckoo wasps present a higher proportion of alkenes compared with linear alkanes, while *P. grandior* possess more linear alkanes than alkenes (as their hosts do) [11,17]. Hence, *P. grandior* seems to have a CHC profile more similar to its hosts than to other, more closely related, cuckoo wasp species.

As in other studied cuckoo wasp species [11,17], chemical camouflage (CHC matching through host CHC acquisition by the parasite) seems unlikely to explain the similarities found between *P. grandior* and *Bembix* spp., since cuckoo wasps have almost no chance to absorb chemicals from hosts or host nests during the short period of nest invasion. Moreover, a camouflage strategy would lead to the absorption of the entire CHC profile of the host. However, *P. grandior* lacked many compounds found on its hosts. Chemical camouflage was indeed found in most studies on social parasites, where, after nest invasion, colony integration and host odour absorption last for days [15,16].

As expected by its wide host range with at least 10 species of *Bembix* [2,19,20,21,22,23,24,25], the chemical mimicry of *P. grandior* could not be as precise as in other cuckoo wasp species which are either specialised in attacking a single host (*C. mediata* and *P. neglecta*) or have strong preference to one of only two hosts (*H. rutilans*) [11,17]. Our results may resemble more what was previously found in *H. nobile* and its host *C. arenaria* [11], probably because *H. nobile* is known to attack at least four *Cerceris* species [23,53]. Additionally, the lower level of mimicry in *H. nobile* may also depend on the fact that its host species possess a larger interspecific CHC profile differences, compared with the hosts of *H. rutilans* [57]. Our results were also similar to those found by Brandt et al. [58] on the socially parasitic ant, *Temnothorax (*= *Protomognathus) americanus* (Emery, 1895), and its several sympatric host species (*Temnothorax* spp.). As in our study, T. americanus had a CHC profile that appeared to be intermediate between sympatric host species. Similarly, Nash et al. [59] found that the CHC profiles of the socially parasitic caterpillars of *Phengaris *(= Maculinea*) *alcon** (Denis & Schiffermüller, 1775) did not display host specificity when two species of *Myrmica* were available at the same site, and that their profile appeared to be a blend of cues from both hosts.

Our behavioural data support a mimicry strategy, since aggressive behaviours of *Bembix* spp. towards *P. grandior* under experimental conditions were less frequent compared to the frequency of tolerant or avoidance behaviours. A similar low level of aggression was observed in *P. triangulum* towards *H. rutilans* inside real ground nests [11], hence, suggesting that the use of circle tubes is an adequate method to assess the host’s recognition ability to natural enemies. Thus, even such moderate CHC matching with hosts seems sufficient in avoiding strong aggression by hosts and thus in successfully parasitizing host nests. Chemical camouflage and insignificance were also reported to reduce host aggression against parasites (camouflage, e.g., *Polistes* social parasites [9,60]; insignificance, e.g., *Sphecodes* cuckoo bees [30]). We did not find differences in the aggression level among the host species of *P. grandior*, all of them having perhaps a similar low ability to recognise cuckoo wasps in their nests. Similarly, the sweat bee *Lasioglossum malachurum* (Kirby, 1802) is pacific towards the cuckoo bee *S. monilicornis* in circle-tubes, but attacks its parasite outside its nests [3,30]. The similarity in aggression level towards *P. grandior* correlates with the similar chemical distances between *P. grandior* and all of its hosts. Interestingly, however, *B. merceti* had the lowest (null) parasitism rate and showed the lowest (null) aggression level towards the cuckoo wasp. This fits to the scenario of an arm-race expecting that lowering costs for hosts lead to lower recognition ability in their hosts [32]. Further data are needed to understand whether *P. grandior* prevalence is really acting as a driving force for increasing recognition abilities in more heavily attacked host species, and to test whether under such circumstances a higher precision of parasite mimicry appears.

We did not find evidence that *P. grandior* evolved chemical insignificance in addition to chemical mimicry. Mimicry and insignificance may co-occur in parasitic insects, since the latter adds concealment and further decreases recognition cues. Chemical insignificance was previously reported among many social parasitic Hymenoptera at the invasion stage, i.e., prior to colony integration and hence camouflage, including obligate social inquiline bumblebees (*Bombus*) [61], socially parasitic ants [15] and socially parasitic wasps [9]. Insignificance was also recently found in solitary cuckoo bees in the genus *Sphecodes* [30]. Chemical insignificance can be achieved by reducing the CHC profile complexity. For example, the cuckoo bee *Sphecodes molinilicornis* (Kirby, 1802) and the velvet ant *Mutilla europea* Linnaeus, 1758, and (before host colony invasion) socially parasitic *Polistes* wasps and socially parasitic ants tend to have a lower number of CHCs than their hosts (halictid bees, *Polistes* wasps and ants, respectively) [6,12,15,30,62]. Furthermore, chemical insignificance may be also achieved by reducing the amount of hydrocarbons [12,58,63]. In addition, chemically insignificant profiles have often a reduced number of substances or even lack entire substance classes important for host recognition (e.g., alkenes) [15,30,64]. None of these strategies could be revealed in *P. grandior*: the CHC profile of the cuckoo wasp has a number of compounds and a total CHCs amount that fall in the range observed of their hosts, and possess abundant alkenes (important for recognition). This is in accordance with the results of a study on the cuckoo wasps *C. mediata* and *P. neglecta* [17], but is in contrast to what was found in another cuckoo wasp, *H. rutilans*, where insignificance (as a reduction of CHCs amount) co-occurred with mimicry [65].

It has been proposed that the outcome of an arms race between parasites and their hosts is the specialization of the parasite to just one host species [4,66], given that a successful parasite requires specialised adaptations to host recognition [6,9,67]. Thus, generalist parasites may be expected to specialise to a single host species at a local scale (local adaptation). This was shown in some socially parasitic ants. For example, both Torres et al. [29] and Bauer et al. [68] found that slave-making ants (*Polyergus mexicanus* Forel, 1899 and *Harpagoxenus sublaevis* (Nylander, 1849), respectively) attack almost exclusively only one of the sympatric ant host species. These locally specialised parasites can be easily distinguished by their different CHC profiles. Similar strategies were also revealed in non-hymenopteran parasites of Hymenoptera hosts. For example, Casacci et al. [28] found that parasitic caterpillars (*Phengaris* (=*Maculinea*) *rebeli* (Hirschke, 1904)) show local variations in host (*Myrmica* ants) specificity, which are consistent with CHC similarities between hosts and parasites at different sites. Local adaptations in chemical deceptive signals were also detected in the parasitic beetle *Meloe franciscanus* Van Dyke, 1928 attacking two allopatric populations of *Habropoda* solitary bees [27]. However, we did not find evidence for local adaptation in *P. grandior*. Indeed, chemical distances between the cuckoo wasp and its hosts were similar. Thus, this cuckoo wasp does not seem to locally adapt by increasing CHC matching with one of the sympatric available hosts. This could be at least partially explained by the rate of parasitism by *P. grandior*, which was not very different between host species.

## 5. Conclusions

Our study investigated the chemical strategy adopted by a generalist chrysidid cuckoo wasp that can chose between more than one host species in a population. We tested two hypotheses: first, *P. grandior* shows a weak chemical mimicry to all its hosts (possibly in conjunction with a reduction of chemical cues in the parasite), and second, the cuckoo wasp shows a local adaptation with a more precise chemical mimicry to one of the available hosts. We found support for the first hypothesis: the low specialization in host use of *P. grandior* lead to a low-level, albeit appreciable chemical mimicry allowing it to successfully attack any of its available host species. A large comparative study is needed to finally test the hypothesis that host range in cuckoo wasps effectively correlates with the precision of chemical mimicry, as previously found in other insect brood parasites of Hymenoptera [69].

## Supplementary Materials

The following are available online at https://www.mdpi.com/2075-4450/11/2/136/s1, Table S1: Raw behavioural data, Table S2: Raw chemical data.

## Appendix A

**Table A1:** Mean value ± standard error of the relative peak area (%) of substances (only those that make up >0.01% per individual and are present in at least half of the group, see Methods) in the cuticular hydrocarbon profiles of the five studied species. The retention index (RI) for each peak is shown.

Substance	RI	*B. sinuata*	*B. zonata*	*B. merceti*	*P. grandior*	*S. continuus*
C20	2000	0.01 ± 0.01	0.03 ± 0.01	0.03 ± 0.00	0.02 ± 0.00	0.00
C21en1	2073	0.00	0.24 ± 0.19	0.00	0.01 ± 0.01	0.00
C21en2	2079	0.01 ± 0.01	0.02 ± 0.02	0.00	0.00	0.00
C21	2100	0.07 ± 0.02	1.73 ± 0.94	0.04 ± 0.01	0.25 ± 0.10	0.00
C22en1	2172	0.00	0.05 ± 0.04	0.02 ± 0.02	0.00	0.00
C22	2200	0.06 ± 0.04	0.07 ± 0.03	0.05 ± 0.00	0.06 ± 0.01	0.00
C23en1	2271	0.76 ± 0.10	0.60 ± 0.44	0.07 ± 0.02	0.00	0.00
C23en2	2275	0.00	1.87 ± 1.77	0.02 ± 0.01	0.10 ± 0.04	0.00
C23en3	2278	0.13 ± 0.04	0.14 ± 0.04	0.04 ± 0.01	0.10 ± 0.05	0.00
C23en5	2292	0.04 ± 0.02	0.02 ± 0.01	0.06 ± 0.01	0.00	0.00
C23	2300	11.66 ± 0.40	11.68 ± 1.74	3.11 ± 0.22	1.54 ± 0.41	0.28 ± 0.04
11,9mC23	2333	0.04 ± 0.03	0.01 ± 0.01	0.02 ± 0.01	0.01 ± 0.01	0.01 ± 0.01
5mC23	2348	0.02 ± 0.01	0.00	0.14 ± 0.01	0.01 ± 0.01	0.01 ± 0.01
3mC23/C24en1	2373	0.74 ± 0.06	0.00	0.03 ± 0.01	0.02 ± 0.01	0.02 ± 0.01
C24	2400	0.58 ± 0.02	0.32 ± 0.12	0.18 ± 0.01	0.12 ± 0.02	0.08 ± 0.01
C25dien3	2458	0.00	0.00	0.03 ± 0.01	0.00	0.00
C25dien4	2463	0.00	0.00	0.05 ± 0.01	0.00	0.00
C25en1	2469	0.00	1.21 ± 0.38	1.18 ± 0.36	0.05 ± 0.05	0.00
C25en2	2473	0.00	0.73 ± 0.73	0.00	0.11 ± 0.04	0.13 ± 0.05
C25en3	2477	27.17 ± 1.38	0.26 ± 0.08	0.38 ± 0.10	0.00	0.00
C25en4	2481	0.00	0.37 ± 0.15	0.93 ± 0.11	0.19 ± 0.07	0.00
C25en5	2485	0.00	0.00	0.00	0.00	0.01 ± 0.01
C25en6	2492	0.00	0.00	0.65 ± 0.09	0.00	0.00
C25	2500	12.70 ± 0.42	10.45 ± 1.33	5.57 ± 0.31	2.49 ± 0.32	2.08 ± 0.55
13,11,9mC25	2531	0.07 ± 0.05	0.03 ± 0.01	0.71 ± 0.08	0.03 ± 0.01	0.40 ± 0.16
7mC25	2538	0.00	0.00	0.00	0.03 ± 0.01	0.21 ± 0.11
5mC25	2549	0.00	0.01 ± 0.01	0.30 ± 0.03	0.28 ± 0.08	0.19 ± 0.06
3mC25 / C26en2	2574	1.07 ± 0.10	0.07 ± 0.02	0.12 ± 0.02	0.06 ± 0.03	0.91 ± 0.33
C26en3	2582	0.00	0.01 ± 0.01	0.00	0.08 ± 0.03	0.00
5,15dimC25/C26en3	2582	0.00	0.00	0.27 ± 0.02	0.00	0.00
5,9/5,11/5,13/5,15dimC25	2585	0.00	0.00	0.00	0.00	0.17 ± 0.08
C26en4	2593	0.00	0.00	0.05 ± 0.01	0.00	0.00
C26	2600	0.29 ± 0.02	0.54 ± 0.07	0.89 ± 0.03	0.81 ± 0.13	0.66 ± 0.11
3,7dimC25	2612	0.00	0.00	0.00	0.00	0.26 ± 0.11
13,12,11,10,9mC26	2632	0.00	0.00	0.00	0.00	0.37 ± 0.10
8,10dimC26	2641	0.00	0.00	0.00	0.00	0.18 ± 0.09
6mC26	2652	0.00	0.00	0.00	0.00	0.02 ± 0.01
C27dien1	2653	0.00	0.06 ± 0.04	0.14 ± 0.01	0.00	0.00
5mC26	2657	0.00	0.00	0.00	0.00	0.07 ± 0.02
C27dien	2660	0.00	0.01 ± 0.01	0.24 ± 0.02	0.00	0.00
4mC26	2663	0.00	0.00	0.00	0.00	0.06 ± 0.01
C27en1	2665	0.00	0.82 ± 0.30	0.47 ± 0.04	0.00	0.00
C27en2	2669	0.00	0.30 ± 0.19	0.77 ± 0.25	1.04 ± 0.41	0.00
C27en3	2672	0.00	0.00	0.93 ± 0.37	0.19 ± 0.08	2.11 ± 0.70
C27en4	2676	14.39 ± 1.09	0.76 ± 0.31	1.02 ± 0.05	0.72 ± 0.44	0.00
C27en5	2680	0.00	0.34 ± 0.11	4.23 ± 0.42	1.85 ± 0.67	0.00
C27en6	2691	0.00	0.00	2.61 ± 0.29	0.00	0.11 ± 0.07
4,8/4,10/4,12dimC26	2693	0.00	0.00	0.00	0.00	0.06 ± 0.02
C27	2700	4.54 ± 0.27	18.52 ± 2.89	29.35 ± 0.97	22.53 ± 1.89	14.72 ± 2.34
13,11,9mC27	2731	0.06 0.04	0.02 ± 0.02	0.87 ± 0.10	0.31 ± 0.07	9.97 ± 1.69
7mC27	2737	0.00	0.00	0.01 ± 0.01	0.03 ± 0.02	1.86 ± 0.31
5mC27	2748	0.00	0.02 ± 0.02	0.29 ± 0.05	0.08 ± 0.03	2.70 ± 0.62
9,13/9,15/9,17dimC27	2764	0.00	0.00	0.00	0.00	0.16 ± 0.07
C28en1	2766	0.00	0.09 ± 0.03	0.01 ± 0.01	0.00	0.00
3mC27/C28en2	2773	0.28 ± 0.05	0.25 ± 0.05	0.22 ± 0.03	1.63 ± 0.17	11.31 ± 0.97
5,17dimC27/C28en3	2779	0.00	0.00	0.23 ± 0.03	0.00	0.00
5,15dimC27	2783	0.00	0.00	0.00	0.00	3.39 ± 0.87
C28en4	2794	0.00	0.00	0.09 ± 0.01	0.00	0.00
C28	2800	0.62 ± 0.03	0.82 ± 0.08	1.19 ± 0.02	0.89 ± 0.19	1.78 ± 0.25
3,15dimC27	2807	0.00	0.00	0.00	0.00	0.58 ± 0.24
3,7dimC27	2810	0.00	0.00	0.00	0.00	1.35 ± 0.27
14,13,12,11,10mC28	2829	0.00	0.00	0.00	0.00	0.92 ± 0.30
8,12/8,14/8,16dimC28	2838	0.00	0.00	0.00	0.00	0.79 ± 0.25
6mC28	2847	0.00	0.00	0.00	0.00	0.15 ± 0.02
C29dien2	2849	0.00	0.25 ± 0.18	0.07 ± 0.01	0.04 ± 0.01	0.00
5mC28	2853	0.00	0.00	0.00	0.00	0.15 ± 0.03
C29dien3	2855	0.00	0.11 ± 0.09	0.27 ± 0.04	0.09 ± 0.05	0.00
4mC28	2861	0.00	0.00	0.00	0.00	0.28 ± 0.08
C29dien4	2865	0.00	0.00	1.33 ± 0.09	0.29 ± 0.12	0.00
C29en2	2866	0.00	3.88 ± 1.28	0.00	0.00	0.00
3mC28/C29en4	2875	3.87 ± 0.46	3.29 ± 1.09	2.96 ± 0.23	36.72 ± 2.40	2.35 ± 0.39
C29en5	2878	0.00	0.46 ± 0.29	1.27 ± 0.09	0.00	0.00
C29en6	2882	0.00	0.34 ± 0.24	1.78 ± 0.24	3.39 ± 0.98	0.00
C29en7	2893	0.00	0.00	2.93 ± 0.37	0.00	0.00
C29	2900	12.57 ± 0.83	16.97 ± 1.74	23.97 ± 0.79	12.91 ± 2.68	13.12 ± 1.06
15,13,11,9mC29	2929	0.11 ± 0.07	0.00	0.35 ± 0.05	0.13 ± 0.05	9.01 ± 3.68
7mC29	2945	0.00	0.00	0.00	0.00	1.07 ± 0.25
5mC29	2947	0.00	0.03 ± 0.03	0.48 ± 0.07	0.04 ± 0.03	2.33 ± 0.44
7,11/7,15/7,17dimC29	2969	0.00	0.00	0.00	0.00	0.62 ± 0.25
3mC29/C30en1	2973	0.00	0.30 ± 0.19	0.10 ± 0.02	0.99 ±10	3.97 ± 2.04
C30en2	2976	0.14 ± 0.04	0.13 ± 0.08	0.00	0.00	0.00
5,17dimC29/C30en3	2977	0.00	0.00	0.09 ± 0.03	0.00	0.00
5,13/5,15/5,17dimC29	2982	0.00	0.00	0.00	0.00	2.30 ± 0.80
C30	3000	0.57 ± 0.05	0.41 ± 0.04	0.40 ± 0.04	0.13 ± 0.04	1.05 ± 0.16
3,7dimC29	3020	0.00	0.00	0.00	0.00	0.29 ± 0.10
15,14,13mC30	3030	0.00	0.00	0.00	0.00	0.13 ± 0.04
6mC30	3046	0.00	0.00	0.00	0.00	0.08 ± 0.03
C31dien2	3047	0.00	0.98 ± 0.54	0.00	0.00	0.00
C31dien3	3052	0.00	0.65 ± 0.53	0.05 ± 0.02	0.01 ± 0.01	0.00
C31dien4	3060	0.00	0.00	0.06 ± 0.03	0.04 ± 0.04	0.00
C31en2	3069	0.00	8.26 ± 2.55	0.04 ± 0.04	0.71 ± 0.15	0.00
C31en3	3077	0.86 ± 0.11	1.96 ± 1.29	0.71 ± 0.05	6.72 ± 1.39	0.39 ± 0.24
C31en4	3085	0.00	0.00	0.28 ± 0.02	0.11 ± 0.11	0.00
C31	3100	5.89 ± 0.76	4.03 ± 1.04	5.04 ± 0.42	1.35 ± 0.36	1.95 ± 0.37
15,13,11mC31	3228	0.12 ± 0.08	0.00	0.01 ± 0.01	0.07 ± 0.03	1.59 ± 0.91
7mC31	3142	0.00	0.00	0.00	0.00	0.09 ± 0.03
5mC31	3147	0.00	0.00	0.02 ± 0.01	0.00	0.24 ± 0.07
C32en1	3165	0.00	0.27 ± 0.09	0.00	0.00	0.00
3mC31/C32en2	3172	0.00	0.00	0.01 ± 0.01	0.00	0.50 ± 0.13
C32	3200	0.11 ± 0.02	0.00	0.02 ± 0.01	0.00	0.11 ± 0.03
C33dien2	3244	0.00	1.44 ± 0.92	0.00	0.00	0.00
C33en2	3266	0.00	2.04 ± 0.53	0.00	0.56 ± 0.18	0.00
C33	3300	0.48 ± 0.08	0.52 ± 0.22	0.13 ± 0.03	0.09 ± 0.01	0.14 ± 0.07
17,15,13,11,9,7mC33	3322	0.00	0.00	0.00	0.00	0.13 ± 0.06
C34en1	3364	0.00	0.63 ± 0.34	0.00	0.00	0.00
C35	3500	0.00	0.57 ± 0.19	0.00	0.00	0.00
